# Supplementary material for: WIP1 mutations suppress DNA damage triggered bypass of the mitotic timer
Source: EMBO J. 2025 Jun 23;44(15):4378–405. doi: 10.1038/s44318-025-00495-0 (PMC12316910; doi:10.1038/s44318-025-00495-0)
Supplement: Supplementary file 1 — Appendix [file 44318_2025_495_MOESM1_ESM.pdf]

Appendix to:

WIP1 mutations suppress DNA damage triggered bypass of the mitotic timer

Tomoaki Sobajima, Luke J. Fulcher, Caleb Batley, Susanna J. Alsop, Jonah Veakins, and Francis A. Barr

Appendix Figure S1. APC/C-Cdh1 directs DNA damaged G2 cells to G1.
Appendix Figure S2. Inhibition of MDM2 triggers G1 cell cycle arrest.

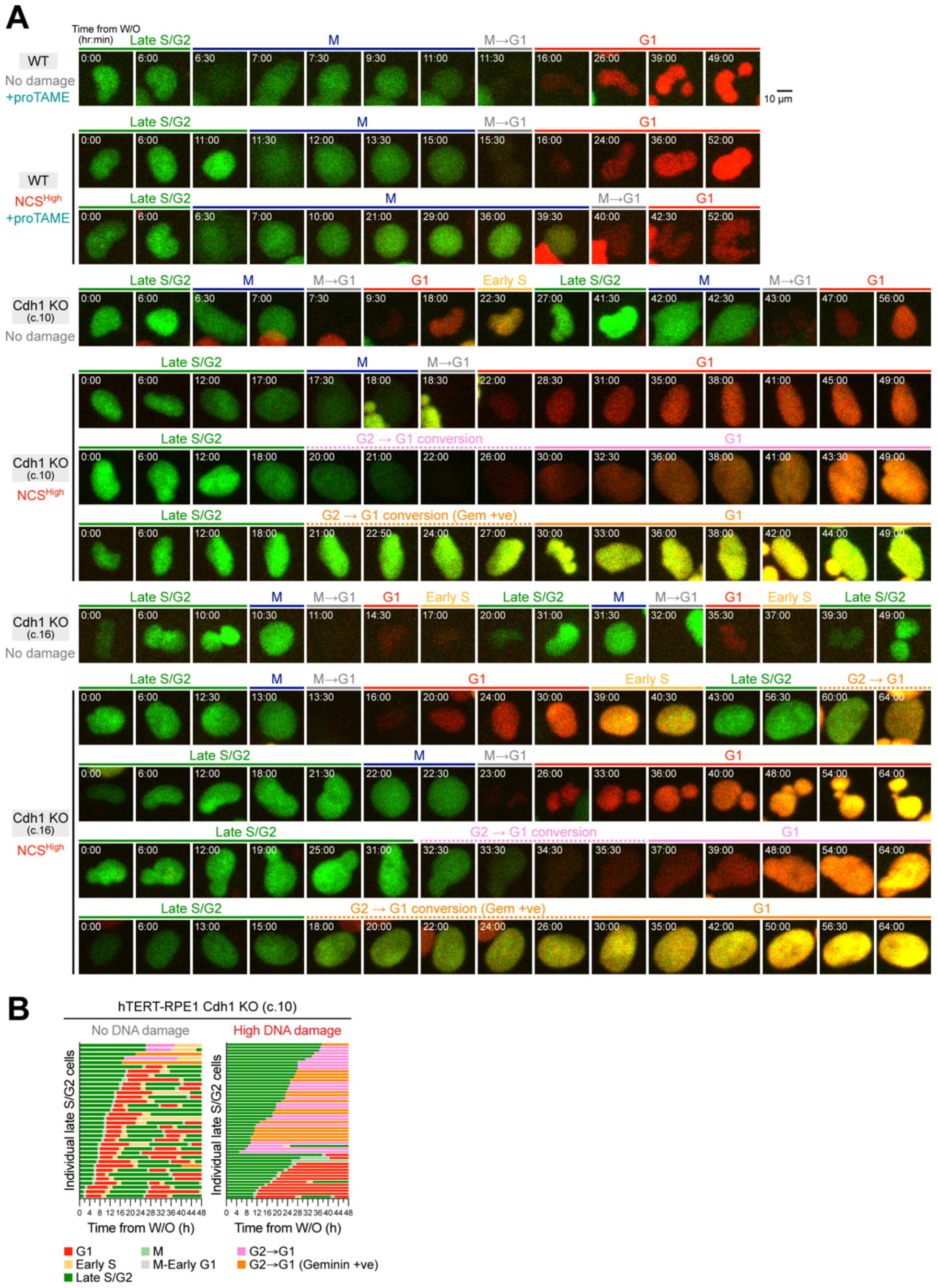

Appendix Figure S1. APC/C-Cdh1 directs DNA damaged G2 cells to G1.

(A) Representative images for the cells described in Figure 4C–4E (n=3 independent experiments) . Scale bar: 10 µm. (B) Cdh1^{KO} hTERT-RPE1 FUCCI clone 10 (c.10) cells were treated with or without high dose NCS for 1 h. Late S/G2 cells were imaged continuously for 2 days. Cell cycle fate is plotted for individual cells. Pooled analyses are shown from 3 independent experiments.

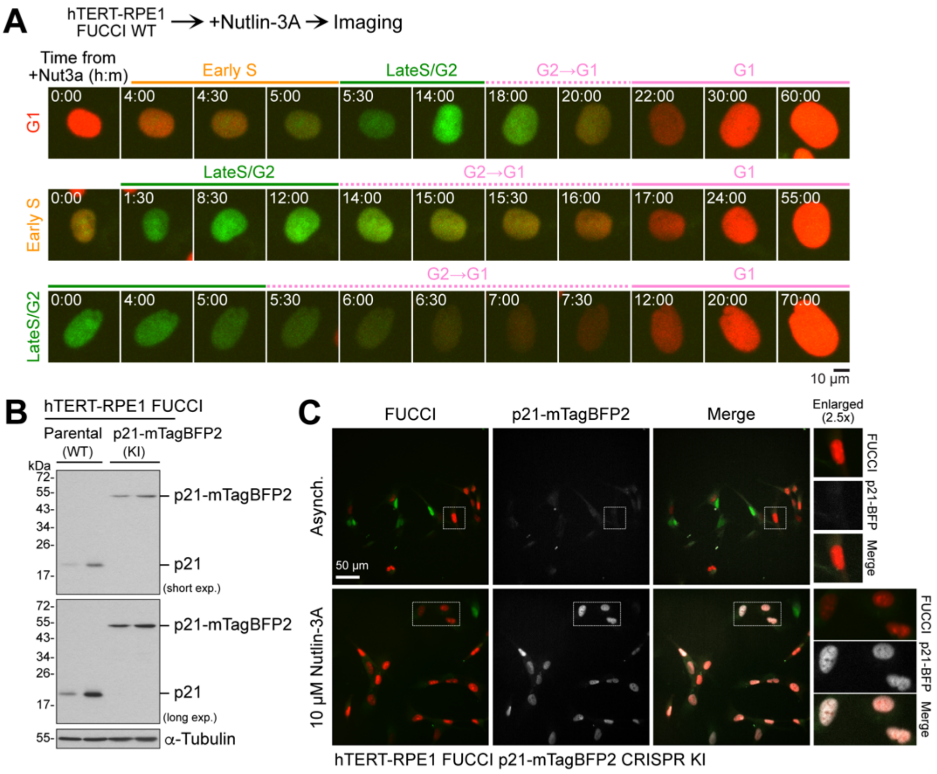

Appendix Figure S2. Inhibition of MDM2 triggers G1 cell cycle arrest.
(A) Representative images for the cells described in Figure 5E (n=3 independent experiments) . Scale bar: 10 µm. (B) Western blot characterisation of the endogenously tagged p21-mTagBFP2 hTERT-RPE1 FUCCI cells used in Figure 5F. Blots are representative of 2 independent experiments. (C) Representative images of p21-mTagBFP2 hTERT-RPE1 FUCCI cells treated with or without Nutlin-3A for 18 h before fixation. Images are representative of 2 independent experiments. Scale bar: 50 µm.
