# Supplementary material for: WIP1 mutations suppress DNA damage triggered bypass of the mitotic timer
Source: EMBO J. 2025 Jun 23;44(15):4378–405. doi: 10.1038/s44318-025-00495-0 (PMC12316910; doi:10.1038/s44318-025-00495-0)
Supplement: Supplementary file 4 — Expanded View Figures [file 44318_2025_495_MOESM4_ESM.pdf]

Expanded View Figures

Figure EV1. Cell cycle response to DNA double strand breaks in hTERT-RPE1 cells.

(**A**) Western blot analysis of the indicated $p53^{KO}$, $p21^{KO}$, $WIP1^{KO}$, and $Cdh1^{KO}$ hTERT-RPE1 FUCCI cell lines (blots are representative of 3 independent experiments). (**B**) Representative images of cells stained for DNA damage markers 53BP1 and γ-H2A.X pS139 analysed in Fig. 1C ($n = 3$ independent experiments). Scale bars: 50 μm and 10 μm (inset). (**C**) $p53^{WT}$ hTERT-RPE1 cells treated with the indicated doses of NCS for 1 h were Western blotted with the antibodies indicated ($n = 3$ independent experiments). (**D**) Levels of γ-H2A-X pS139, p53 and p53 pSer15 from panel (**C**) are plotted (mean ± SEM; $n = 3$ independent experiments). (**E**) Representative images for the cells described in Fig. 1D ($n = 3$ independent experiments). Scale bar: 10 μm.

© The Author(s)

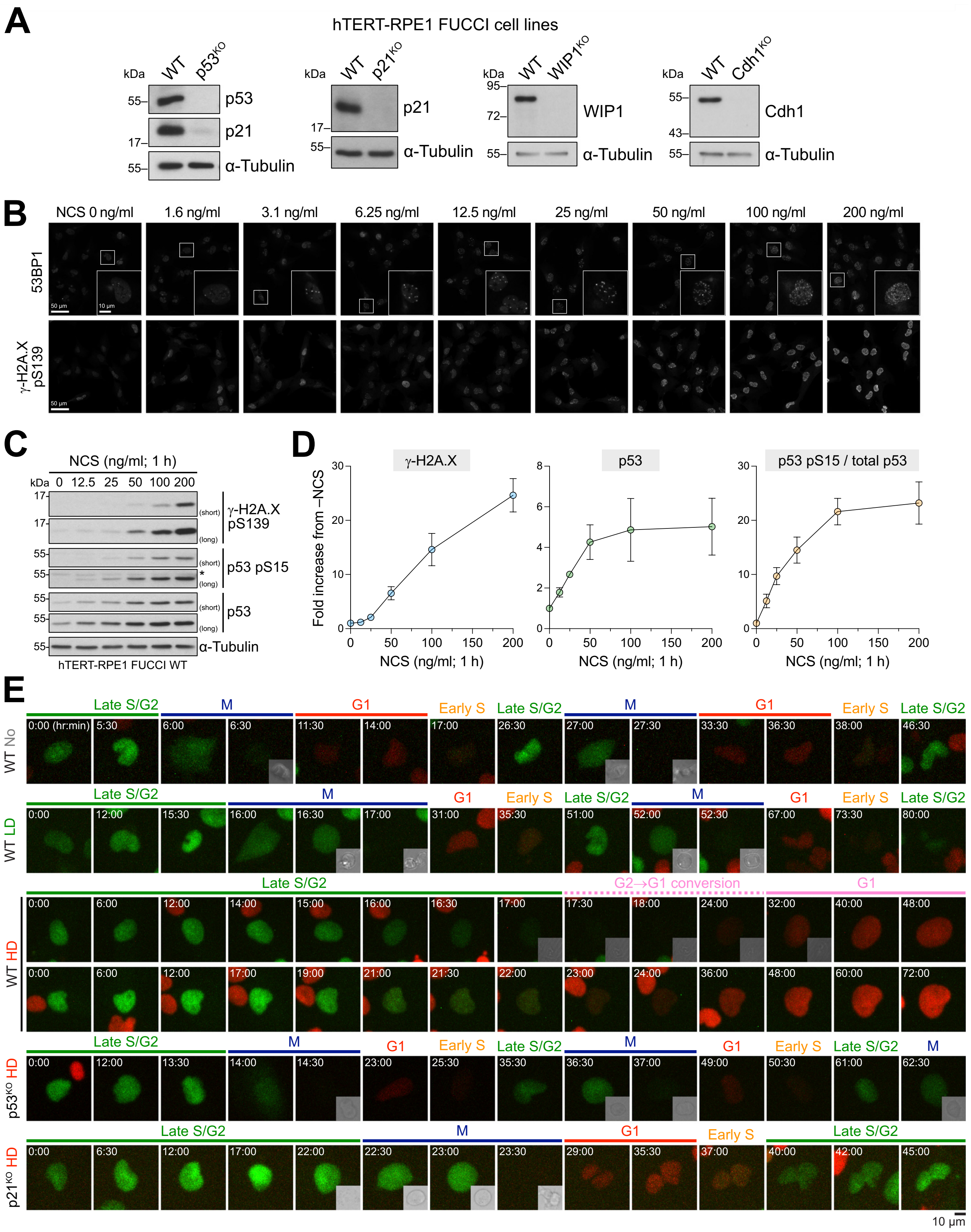
A
hTERT-RPE1 FUCCI cell lines
kDa WT p53KO
p53
p21
α-Tubulin
kDa WT p21KO
p21
α-Tubulin
kDa WT WIP1KO
WIP1
α-Tubulin
kDa WT Cdh1KO
Cdh1
α-Tubulin
B
NCS 0 ng/ml 1.6 ng/ml 3.1 ng/ml 6.25 ng/ml 12.5 ng/ml 25 ng/ml 50 ng/ml 100 ng/ml 200 ng/ml
53BP1
γ-H2A.X pS139
C
NCS (ng/ml; 1 h)
kDa 0 12.5 25 50 100 200
γ-H2A.X pS139
p53 pS15
p53
α-Tubulin
hTERT-RPE1 FUCCI WT
D
γ-H2A.X
p53
p53 pS15 / total p53
Fold increase from −NCS
NCS (ng/ml; 1 h)
E
WT No
WT LD
WT HD
p53KO HD
p21KO HD
Late S/G2
M
G1
Early S
G2→G1 conversion
10 μm

 © The Author(s)

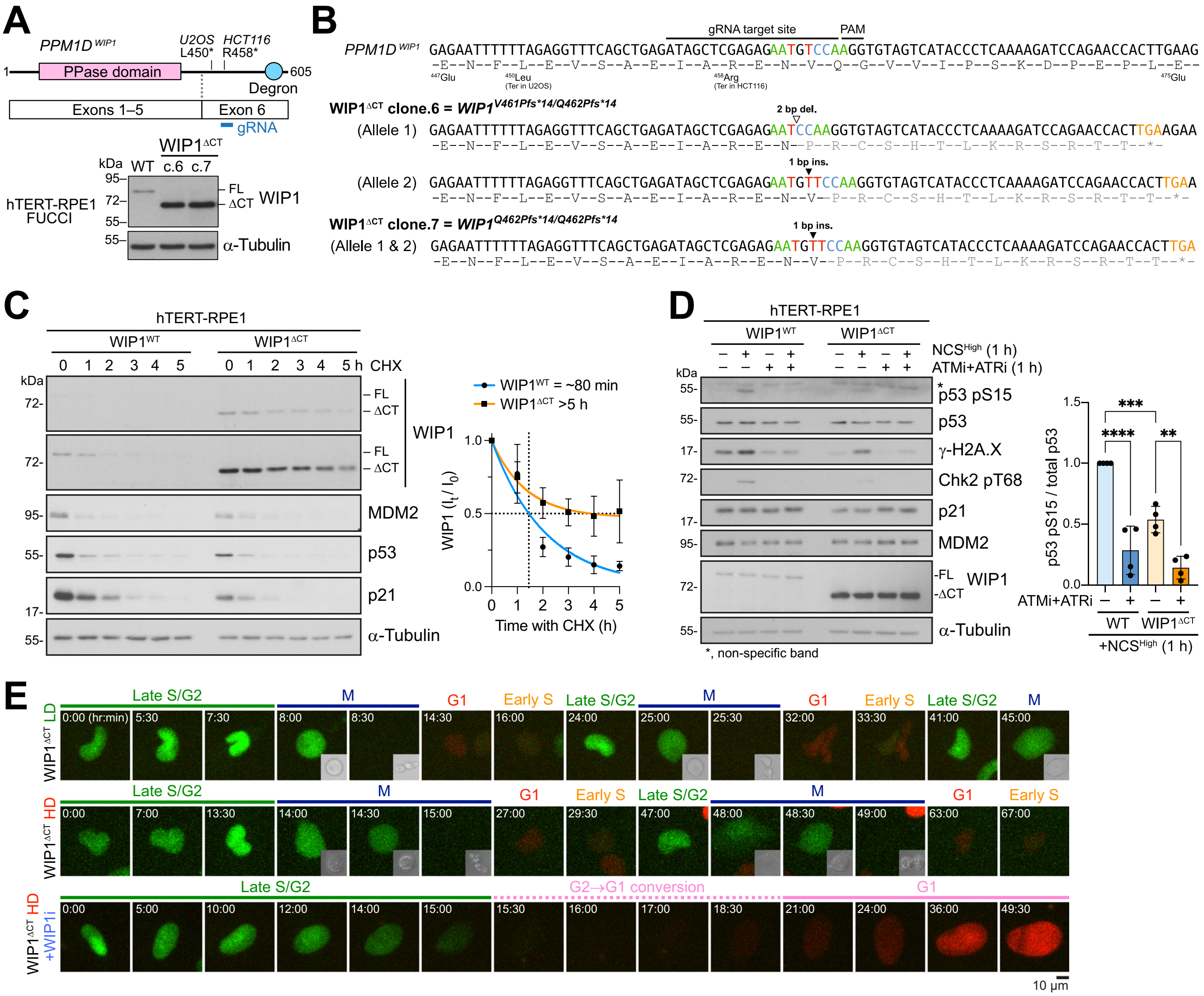

Figure EV2. Generation and characterisation of hTERT-RPE1 WIP1$^{\Delta CT}$ cells.

(**A**) Schematic detailing the CRISPR/Cas9 gene editing strategy used to obtain WIP1$^{\Delta CT}$ cells (top). Western blot validation of WIP1$^{\Delta CT}$ hTERT-RPE1 FUCCI cells (bottom; blots representative of 3 independent experiments). (**B**) DNA sequencing demonstrating successful WIP1$^{\Delta CT}$ generation in hTERT-RPE1 FUCCI cells ($n = 1$). (**C**) WIP1^{WT} and WIP1$^{\Delta CT}$ hTERT-RPE1 FUCCI cells were treated with cycloheximide (CHX) for the indicated times and then Western blotted with the indicated antibodies (left). The relative half-lives of full-length WIP1 and WIP1$^{\Delta CT}$ are plotted (right; mean ± SEM; $n = 3$ independent experiments). (**D**) WIP1^{WT} and WIP1$^{\Delta CT}$ hTERT-RPE1 FUCCI cells were treated with NCS and ATM/ATR inhibitors as shown for 1 h before lysis. Samples were subjected to immunoblotting with the indicated antibodies (left). The ratio of p53 pSer15/total p53 is plotted for the different conditions (right; mean ± SD; $n = 4$ independent experiments). Statistical significance was analysed using an ordinary one-way ANOVA with Tukey's multiple comparisons test (**$P < 0.01$; ***$P < 0.001$; ****$P < 0.0001$). (**E**) Representative images for the cells described in Fig. 2H ($n = 3$ independent experiments). Scale bar: 10 µm. All P values are listed in Dataset EV1.

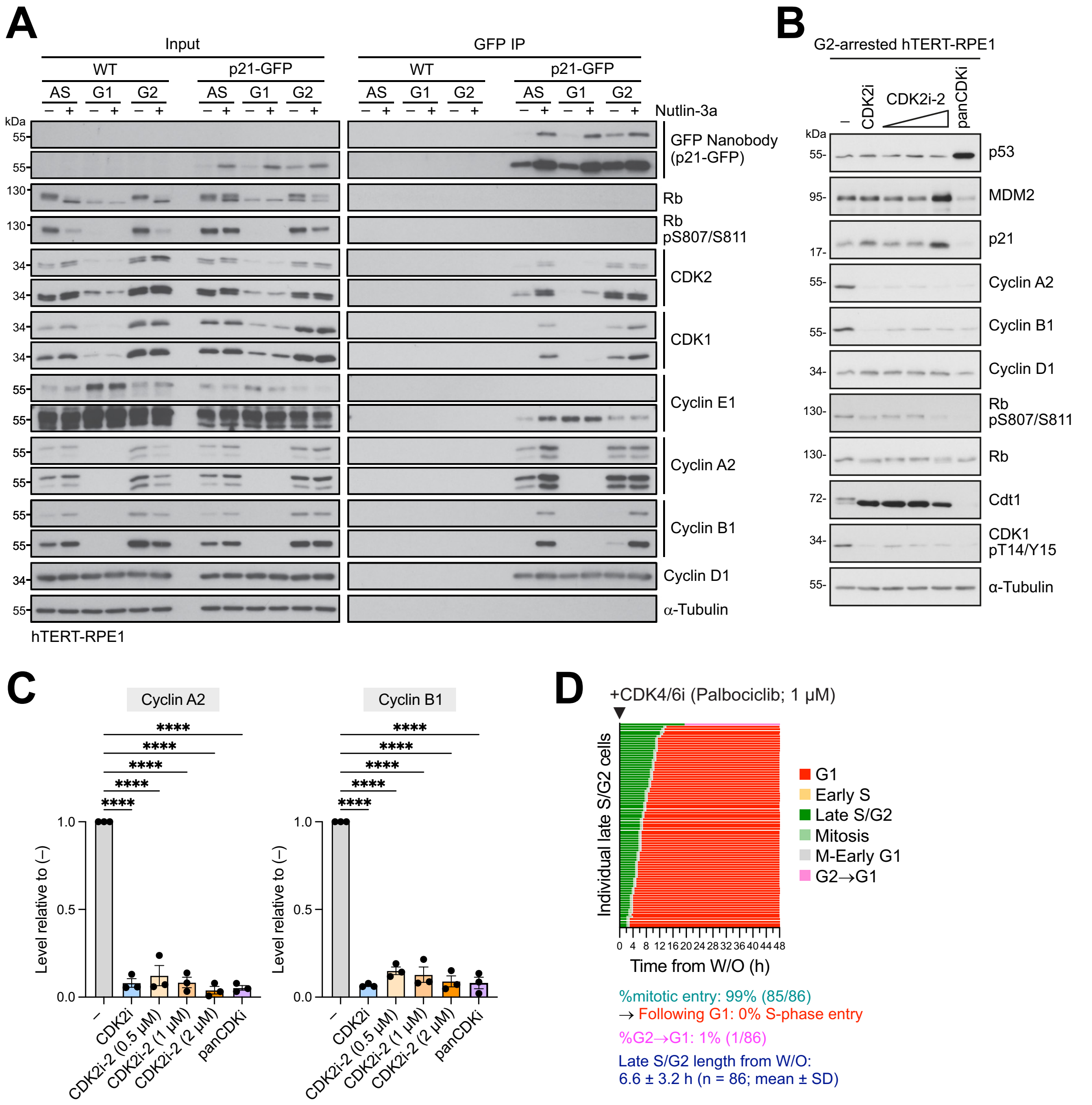

Figure EV3. Differential targeting of CDK-cyclin complexes by p21 in G1 and G2.

(**A**) Wild-type and p21-GFP hTERT-RPE1 cells were arrested in G1 with CDK4/6i or in G2 with CDK1i for 18 h. Asynchronous (AS) cells were included as a control. Cells were treated with or without Nutlin-3A for 6 h prior to lysis, and proteins were isolated from the lysates by GFP-TRAP beads (GFP IP). Input and IP extracts were subjected to immunoblotting with the indicated antibodies ($n = 2$ independent experiments). (**B**) Wild-type hTERT-RPE1 FUCCI cells were synchronised in G2 with CDK1 inhibitor for 18 h, treated with 100 nM of the CDK2 inhibitor PF-06873600 (CDK2i), 500 nM, 1 µM or 2 µM (left to right) of the alternative CDK2 inhibitor INX-315 (CDK2i-2), or 5 µM of the pan CDK inhibitor Flavopiridol (panCDKi) or DMSO (–) for 8 h, and Western blotted for cell cycle markers. (**C**) Cyclin A2 and B1 levels after 8 h treatment as in (**B**) are plotted for each condition (mean ± SEM; $n = 3$ independent experiments). Statistical significance was analysed using a one-way ANOVA with Dunnett's multiple comparisons test (****$P < 0.0001$). (**D**) Wild-type hTERT-RPE1 FUCCI cells were treated with the CDK4/6 inhibitor Palbociclib (CDK4/6i) for 1 h before late S/G2 cells were imaged continuously for 2 days. Cell cycle fate is plotted for individual cells. Pooled analyses are shown from 3 independent experiments. All *P* values are listed in Dataset EV1.

 © The Author(s)

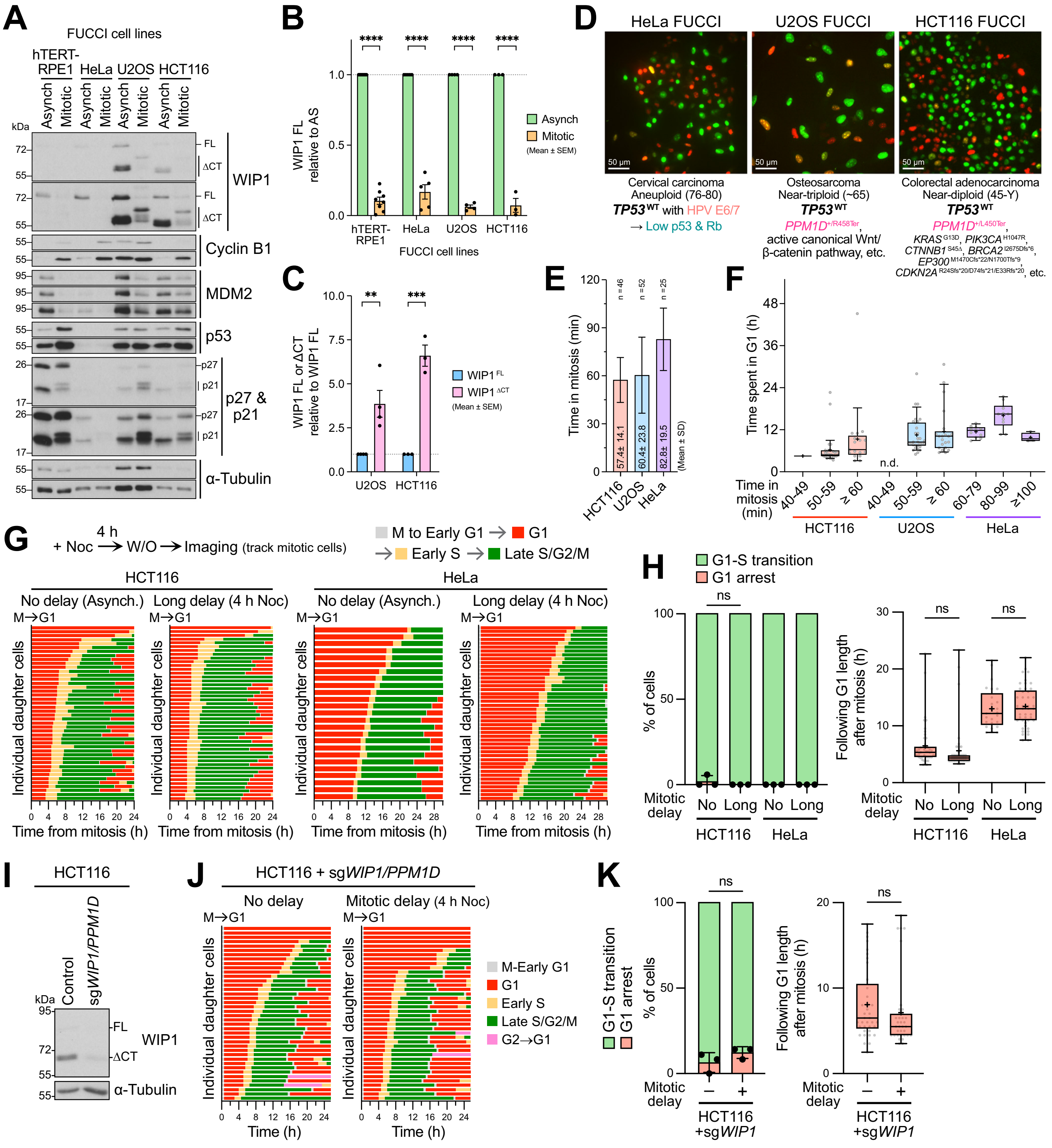
A
FUCCI cell lines
hTERT-RPE1
HeLa
U2OS
HCT116
Asynch
Mitotic
WIP1
Cyclin B1
MDM2
p53
p27 & p21
α-Tubulin
B
WIP1 FL relative to AS
Asynch
Mitotic
(Mean ± SEM)
FUCCI cell lines
C
WIP1 FL or ΔCT relative to WIP1 FL
D
HeLa FUCCI
U2OS FUCCI
HCT116 FUCCI
Cervical carcinoma
Aneuploid (76-80)
TP53WT with HPV E6/7
→ Low p53 & Rb
Osteosarcoma
Near-triploid (~65)
TP53WT
active canonical Wnt/β-catenin pathway, etc.
Colorectal adenocarcinoma
Near-diploid (45-Y)
TP53WT
E
Time in mitosis (min)
57.4± 14.1
60.4± 23.8
82.8± 19.5
(Mean ± SD)
F
Time spent in G1 (h)
Time in mitosis (min)
G
+ Noc → W/O → Imaging (track mitotic cells)
M to Early G1
G1
Early S
Late S/G2/M
No delay (Asynch.)
Long delay (4 h Noc)
Individual daughter cells
Time from mitosis (h)
H
G1-S transition
G1 arrest
% of cells
Following G1 length after mitosis (h)
Mitotic delay
I
Control
sgWIP1/PPM1D
J
HCT116 + sgWIP1/PPM1D
No delay
Mitotic delay (4 h Noc)
M-Early G1
G2→G1
Time (h)
K
HCT116 +sgWIP1

Figure EV4. Analysis of the mitotic timer in tumour cell lines with WIP1 mutations or lacking p53 function.

(**A**) hTERT-RPE1, HeLa, U2OS and HCT116 FUCCI cells were arrested in mitosis for 18 h with nocodazole and western blotted with the indicated antibodies. Asynchronous (AS) cells were included as controls ($n = 3$–8 independent experiments). (**B**) The levels of full-length WIP1 in mitotic arrest conditions relative to AS from (**A**) are plotted (mean ± SEM; $n = 3$–8 independent experiments). Statistical significance was analysed using unpaired two-tailed *t* test. (**C**) Relative levels of full-length WIP1 to the ΔCT fragment are plotted for U2OS and HCT116 cells described in (**A**) (mean ± SEM; $n = 3$–4 independent experiments). Statistical significance was analysed using unpaired two-tailed *t* test. (**D**) Representative images of the HeLa, U2OS and HCT116 FUCCI cell lines, with known mutations highlighted underneath. Scale bars: 50 μm. (**E**) Mean time in mitosis in HCT116, U2OS and HeLa FUCCI cells in asynchronous culture is plotted (mean ± SD; pooled analyses shown from 3 independent experiments). (**F**) G1 length for HCT116, U2OS and HeLa FUCCI cells are plotted in a box and whiskers plot, categorised based on their time in mitosis (median, 25th and 75th percentiles and whiskers extending to minimum and maximum values; mean (+) for the different conditions; pooled analyses shown from 3 independent experiments). n.d.: no data points could be obtained. (**G**) HCT116 and HeLa FUCCI cells were treated with or without nocodazole to induce mitotic delay (4 h) as indicated. Nocodazole was removed by washing, and the mitotic cells were imaged continuously. Cell cycle fate is plotted for individual cells. Pooled analyses are shown from 3 independent experiments. (**H**) The percentage of cells undergoing the G1/S transition or G1 arrest following the treatments as in (**G**) is plotted (mean ± SD; $n = 3$ independent experiments) (left). Statistical significance was analysed using an unpaired two-tailed *t* test. G1 length following mitosis in cells that entered mitosis following treatments as in (**G**) is plotted for individual cells in a box and whiskers plot (median, 25th and 75th percentiles and whiskers extending to minimum and maximum values, mean (+) for the different conditions; pooled analyses shown from 3 independent experiments) (right). Statistical significance was analysed using an unpaired two-tailed *t* test. (**I**) Western blot showing efficient WIP1 knockout in HCT116 FUCCI cells targeted with WIP1 single guide RNA (sgWIP1/PPM1D) ($n = 1$ experiment). (**J**) HCT116 FUCCI cells described in (**I**) were treated with nocodazole to induce mitotic delay (4 h) or left untreated (No delay). Nocodazole was removed by washing, and the cells then imaged continuously for 24 h. Cell cycle fate is plotted for individual cells. Pooled analyses are shown from 3 independent experiments. (**K**) The percentage of cells undergoing the G1/S transition or G1 arrest after mitotic delay or no delay described in (**J**) is plotted (left) (mean ± SD; $n = 3$ independent experiments). Statistical significance was analysed using an unpaired two-tailed *t* test. G1 length following mitosis in cells that entered mitosis following treatments as in (**J**) is plotted for individual cells in a box and whiskers plot (median, 25th and 75th percentiles and whiskers extending to minimum and maximum values, mean (+) for the different conditions; pooled analyses shown from 3 independent experiments) (right). Statistical significance was analysed using an unpaired two-tailed *t* test. Significance for all experiments: $^{**}P < 0.01$; $^{***}P < 0.001$; $^{****}P < 0.0001$; ns, not significant. All *P* values are listed in Dataset EV1.

 © The Author(s)
